# Supplementary material for: A network-based approach to identify deregulated pathways and drug effects in metabolic syndrome
Source: Nat Commun. 2019 Nov 18;10:5215. doi: 10.1038/s41467-019-13208-z (PMC6861239; doi:10.1038/s41467-019-13208-z)
Supplement: Supplementary file 1 — Supplementary Information [file 41467_2019_13208_MOESM1_ESM.pdf]

Supplementary Information for

A network-based approach to identify deregulated pathways and drug effects in metabolic syndrome

Karla Misselbeck[1,2], Silvia Parolo[1], Francesca Lorenzini[3], Valeria Savoca[3], Lorena Leonardelli[1], Pranami Bora[1], Melissa J. Morine[1], Maria Caterina Mione[3], Enrico Domenici[1,3], Corrado Priami[1,4]

[1] Fondazione The Microsoft Research - University of Trento Centre for Computational and Systems Biology (COSBI), Rovereto (TN), Italy

[2] Department of Mathematics, University of Trento, Povo (TN), Italy

[3] Department of Cellular, Computational and Integrative Biology (CIBIO), University of Trento, Povo (TN), Italy

[4] Department of Computer Science, University of Pisa, Pisa (PI), Italy

Content

Supplementary Note 1: Comparison with related work 2

Supplementary Note 2: Contribution of the different data sources to the final predictions..4

Supplementary Note 3: Evaluation of score effectiveness..5

Supplementary Figures 7

Supplementary Tables..22

Supplementary References 30

Supplementary Note 1: Comparison with related work

In recent years, numerous computational drug repurposing methods have been developed. In particular, network-based *in silico* repositioning strategies have received great attention given the suitability of network data structures to represent biological data in an efficient way. The most commonly adopted network-based computational strategies have been described in recent review articles[1,2].

The available methods can be categorized according to the type of input data they require as well as the type of network used and the computational strategy applied to identify repurposing drug candidates.

With regard to the input data, common strategies are based on gene/protein expression profiles of drugs and diseases, disease symptoms, chemical structures of drugs, reported drug side effects, known drug targets, known drug indications, and/or clinical data related to the drug of interest (from clinical trials and electronic health records)[3].

To describe the interactions among the players of interest, different types of networks can be considered. Protein-protein interaction networks, gene regulatory networks and metabolic networks are commonly used to represent the interactions among the molecular players that constitute a biological system (protein-protein, transcription factor-gene, and metabolite-metabolite interactions). For example, a recent study by Cheng and collaborators identified new drug repurposing candidates for cardiovascular disease mapping both disease genes and drug targets on a protein-protein interaction network[4]. The transcriptomic profiles of drugs and diseases can also be analyzed using network-based approaches. For example, the drug-gene signatures from Connectivity Map[5,6] have been integrated with genes frequently mutated in cancer to identify new drugable targets and anticancer indications for existing drugs[7]. In addition, also information present in genome-scale metabolic models have been successfully integrated in a network-based setting to identify drug targets for the treatment of metabolic disorders[8,9]. Heterogeneous networks constituted by different types of nodes, such as drugs, targets or diseases, can be generated. The analysis of these kinds of networks is commonly used for the prediction of new drug-target interactions[2]. In this case networks with at least two distinct types of nodes are constructed on the basis of known associations and different algorithms are used to predict new interactions.

The prediction of new drug applications or drug-disease associations can be based solely on the network characteristics or exploit other sources of information such as drug structure similarity, target similarity, disease similarity[1].

Inspired by these network-based repurposing studies, we developed a new computational strategy that tries to combine different input data, background networks and similarity measures in an innovative framework.

The background network was built by integrating protein-protein and transcription factor-gene interactions; the latter was demonstrated as particularly suitable to cluster GWAS derived disease genes. A further strength of our study is the tissue-specificity of the generated networks, as it was extensively demonstrated that gene function is frequently related to the tissue and disease considered[10-15].

To obtain a detailed description of the disease pathways, we integrated genetic information derived from GWAS with literature-derived knowledge. GWAS data has already been exploited for computational repurposing[16]. However, to address the problem of distinguishing causal genes from genes located in the same genomic region but not involved in the disease predisposition, we performed a preliminary filtering step based on pathway enrichment analysis, similarly to previous GWAS prioritization studies[17-21]. Moreover, the GWAS results were complemented with literature that captures also non-GWAS findings, such as the results of functional studies.

For the drugs, we combined gene expression profiles with information about the drug target. The network-based setting allowed us integrating this two distinct information using a novel strategy to identify drug modules. Indeed, the module detection is based on the idea that the gene expression perturbation observed in the drug signature profiles is a cascade starting with the drug-target binding. Moreover, the regulatory-network component of our background network supports this reasoning because it describes the transcriptional regulation exerted by transcription factors.

Another aspect that separates our strategy from previously published methods is the combined use of topological network information and semantic similarities to identify repurposing candidates. The two approaches have been used separately by others to identify new disease-drug interactions[22,23]. While the idea to combine network characteristics and functional similarities has already been applied for the detection of network modules[24], the integration of semantic similarities and topological properties between network modules has not yet been applied to identify novel disease-drug relations.

Supplementary Note 2: Contribution of the different data sources to the final predictions

In the pipeline we developed, several data sources are integrated by means of network analysis. Tissue-specific networks, generated by merging protein-protein interaction networks and transcriptional regulatory networks, provided the scaffold of the analysis. GWAS results and text mining results were combined to detect disease relevant network modules (MetSyn modules). Gene set enrichment analysis of GWAS genes identified significant pathways that were the basis for filtering both the GWAS and text mining genes. Specifically, only genes that are part of at least one significant pathway were selected for subsequent analyses. The identification of drug repurposing candidates was based on the definition of a proximity score that connects drug modules and MetSyn modules combining network-based distance and functional similarity.

In the following section, the contribution of the individual system components to the final prediction will be described (GWAS vs. text mining, PPI vs. regulatory network, network-based distance vs. GO functional similarity).

1) GWAS vs. text mining

With our workflow that combines genes derived from GWAS and text mining results, two MetSyn modules were identified in the adipose network, and three MetSyn modules were detected for liver and muscle. If we consider only the genes derived from GWAS results (286 genes, Supplementary Data 3), one out of two MetSyn module in the adipose network, one out of three MetSyn module in the muscle network, and three out of three MetSyn modules in the liver network can be detected. On the other hand, taking into account only genes derived from text mining results (546 genes, Supplementary Data 3), leads to the detection of two out of two MetSyn module in the adipose network, three out of three MetSyn modules in the muscle network, and two out of three MetSyn modules in the liver network. However, the final selection of text mining derived genes is guided by the GWAS results through pathway analysis and thus the comparison of the individual contribution of text mining and GWAS is not straightforward and should be interpreted in the context of the entire methodological framework. Overall, we can observe that the addition of text mining genes allows a more detailed disease characterisation. For example, the inflammation-related MetSyn module in the adipose network would not have been identified if the analysis had been limited to the genes derived solely from GWAS. Moreover, the source (GWAS and text mining) of the MetSyn genes included in the

modules was evaluated and is summarised in Supplementary Table 2. Overall, the text mining genes play a major role for the disease enrichment analysis. In all, except two cases (Liver Modules 1 and 3), text mining genes accounted for the significance of the modules.

2) PPI vs. regulatory network

To evaluate the contribution of the protein-protein and regulatory network interactions, we first evaluated the composition of the complete networks (Supplementary Table 3) and afterwards the composition of the MetSyn modules for both edge and node source (Supplementary Tables 4 and Supplementary Table 5). Overall, we can conclude that the edges and nodes derived from the PPI networks contribute more to the overall network structure. On the level of MetSyn modules, only one module (Liver Module 1) is solely constructed based on edges and nodes from the regulatory network. This relatively small module is related to the modification of chemicals (Reactome pathway: Phase II - Conjugation of compounds).

3) network-based distance vs. GO functional similarity

Finally, we evaluated the contribution of the closest network distance and the GO similarity to the final significant results by testing how many of the significant results remain significant if only the topological aspect or the semantic similarity was considered. With the integrated proximity score, we identified 28, 31 and 50 significant drugs in the adipose, liver and muscle network, respectively.

Overall, for all MetSyn modules the number of significant results increases using the integrated score (Supplementary Figure 4a). The evaluation of the relative contribution of the two aspects showed that the GO similarity score alone identified more significant drugs than the topological score alone (Supplementary Figure 4b). The only module for which the topological score has a greater impact is Module 2 in the muscle network (Supplementary Figure 4).

Supplementary Note 3: Evaluation of score effectiveness

To test the effectiveness of our approach in identifying drugs affecting MetSyn-pathways, we looked at drugs already approved for MetSyn-related traits. We identified them by parsing the indications provided by DrugBank[25] (version 5.1.1) and filtering for the following search-terms:

obesity, hyperglycemia, glycemicontrol, dyslipid(a)emia, high cholesterol, high triglyceride, hyperlipid(a)emia, hypertension, high blood pressure, NIDDM, cholesterol-lowering, antilipemic, statins, lipid-lowering, hypercholesterolemia, and diabetes.

For the “diabetes” search-term, a further refinement was carried out limiting the results to those referring to type 2 diabetes. Moreover, for the adipose tissue, the resulting list was trimmed to those drugs affecting adipose tissue and those for which we were able to generate a drug module in the adipose network (dependent on availability of drug target and signature) (see Supplementary Table 6).

Based on the information provided in DrugBank[25] and ChEMBL[26], six of the resulting ten drugs have an obvious impact on adiposity (bezafibrate, clofibrate, fenofibrate, gemfibrozil, mifepristone, and pioglitazone), while four drugs (atorvastatin, glipizide, lovastatin and repaglinide) did not have a direct connection to adiposity. However, for atrovastatin and lovastatin recent publications suggest their impact also on adiposity[27-29]. The two sulfonylureas drugs glipizide and repaglinide were also kept because of a described effect on adipose tissue via the inhibition of lipolysis[30,31]. For this reason we executed two analyses, one with all the 6 top MetSyn drugs (Analysis A: bezafibrate, clofibrate, fenofibrate, gemfibrozil, mifepristone, and pioglitazone), and an additional one including all ten MetSyn drugs (Analysis B: bezafibrate, clofibrate, fenofibrate, gemfibrozil, mifepristone, pioglitazone, atorvastatin, glipizide, lovastatin and repaglinide).

To evaluate the effectiveness of our scoring system, we tested how many MetSyn drugs our method could identify at different significance thresholds (Supplementary Fig. 5). With our selected significance threshold of 95% we are able to detect half of the MetSyn drugs in both analysis (Analysis A: pioglitazone, mifepristone, and fenofibrate; Analysis B: pioglitazone, mifepristone, fenofibrate, glipizide and lovastatin). This still holds for the threshold of 99% for Analysis A, while for Analysis B the ratio of detected MetSyn drugs decreases because glipizide and lovastatin are no longer significant. Lowering the threshold to 85% we could identify all six top MetSyn drugs, while atorvastatin and repaglinide are only identified at a threshold level of 75% and 50%, respectively.

Supplementary Figures

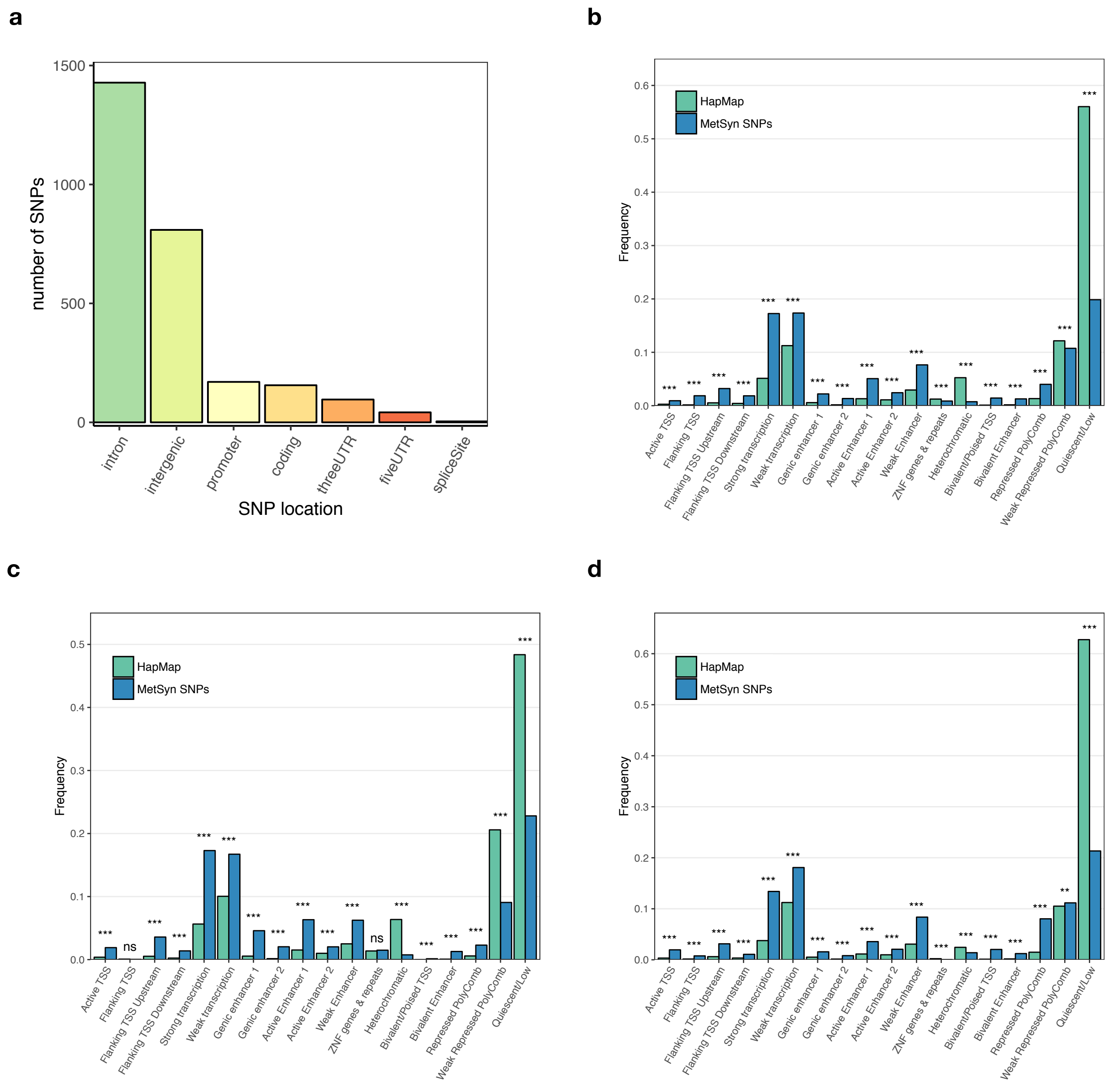

Supplementary Figure 1: Functional annotation of SNPs from GWAS catalog. a Functional annotation of the SNPs extracted from GWAS catalog[32]. SNPs were categorized in one or more of the indicated categories according to their genomic location. The location of the analyzed gene elements was retrieved from the University of California Santa Cruz (UCSC) Known Genes dataset[33]. **b-d** Annotation of SNPs according to their position relative to regulatory regions. The positions of MetSyn-related GWAS SNP and the 18-chromatin-state annotation of a) adipose tissue, b) liver tissue and c) skeletal muscle tissue obtained from the NIH Roadmap Epigenomics project[34] were overlapped to evaluate the regulatory potential of the identified SNPs. The full set SNP of HapMap SNPs was analyzed in the same way for comparison. Fisher exact test was used to test the over-/underrepresentation of GWAS SNPs in regulatory regions. Statistical significance is denoted as follows: ns: not significant (p-value > 0.05), * : 0.01 < p-value <= 0.05, ** : 0.01 < p-value <= 0.001, *** : p-value <0.001.

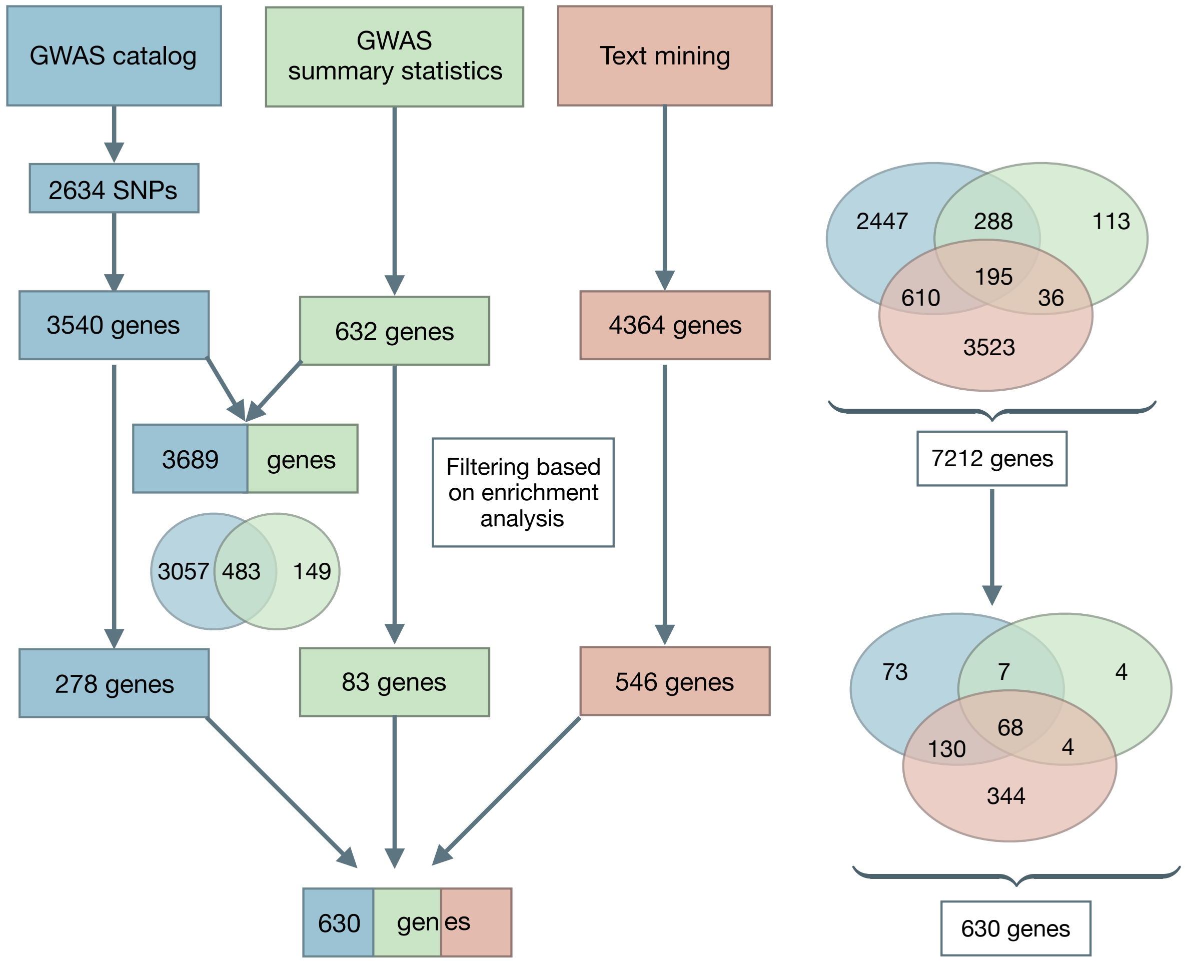

Supplementary Figure 2: Overview of MetSyn genes identification. For each of the three resources (GWAS catalog, GWAS summary statistics and text mining) the number of genes and their intersection is shown.

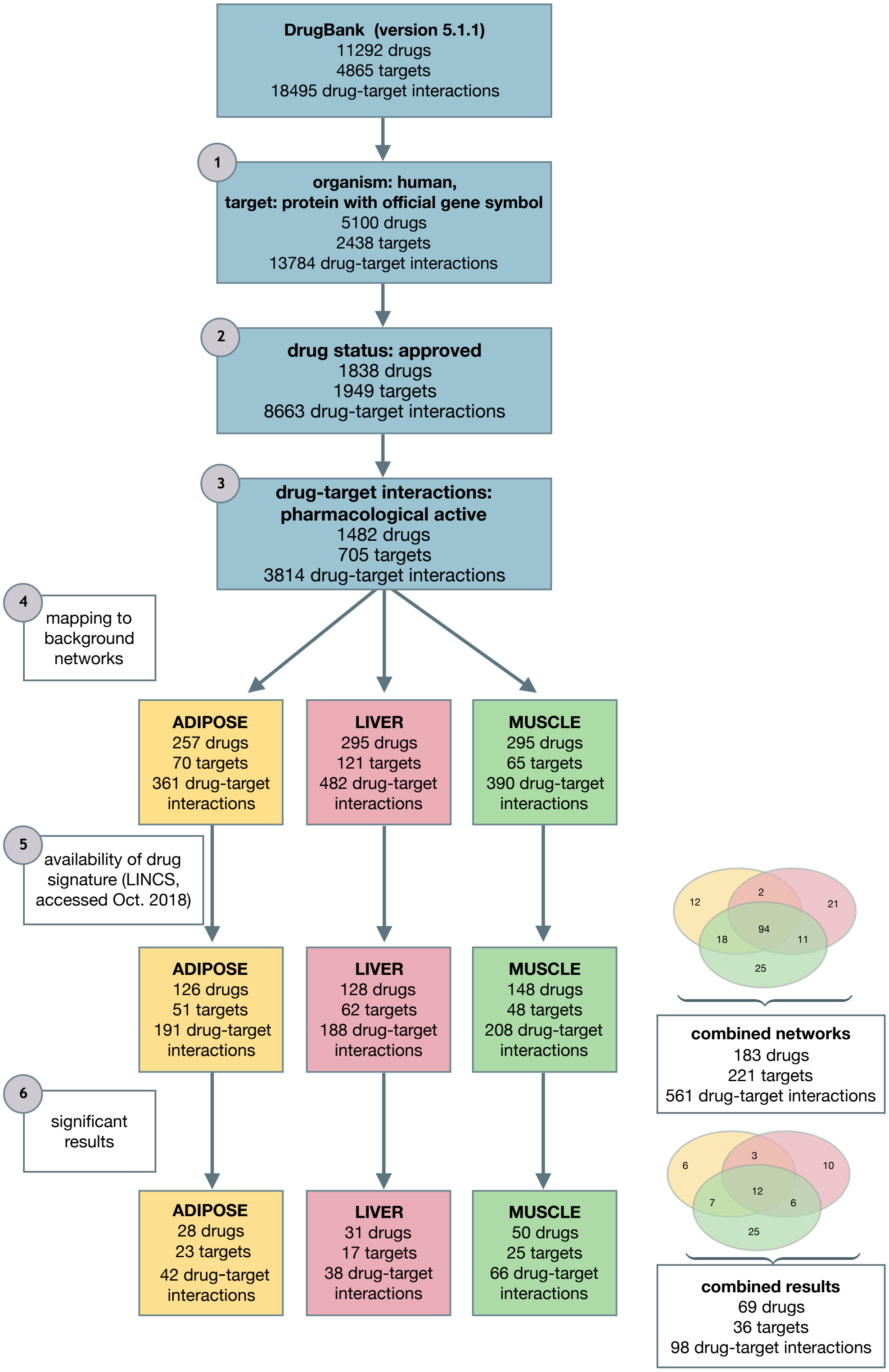
DrugBank (version 5.1.1)
11292 drugs
4865 targets
18495 drug-target interactions
1
organism: human,
target: protein with official gene symbol
5100 drugs
2438 targets
13784 drug-target interactions
2
drug status: approved
1838 drugs
1949 targets
8663 drug-target interactions
3
drug-target interactions:
pharmacological active
1482 drugs
705 targets
3814 drug-target interactions
4
mapping to background networks
ADIPOSE
257 drugs
70 targets
361 drug-target interactions
LIVER
295 drugs
121 targets
482 drug-target interactions
MUSCLE
295 drugs
65 targets
390 drug-target interactions
5
availability of drug signature (LINCS, accessed Oct. 2018)
ADIPOSE
126 drugs
51 targets
191 drug-target interactions
LIVER
128 drugs
62 targets
188 drug-target interactions
MUSCLE
148 drugs
48 targets
208 drug-target interactions
12
2
21
94
18
11
25
combined networks
183 drugs
221 targets
561 drug-target interactions
6
significant results
ADIPOSE
28 drugs
23 targets
42 drug-target interactions
LIVER
31 drugs
17 targets
38 drug-target interactions
MUSCLE
50 drugs
25 targets
66 drug-target interactions
6
3
10
12
7
6
25
combined results
69 drugs
36 targets
98 drug-target interactions

Supplementary Figure 3: Overview of drug selection. The drugs available in DrugBank[25] (version 5.1.1) were filtered according to the following steps: We kept 1) drugs having human as the organisms for which the drug is most effective and whose target(s) could be mapped to official gene symbol(s). 2) approved drugs. 3) targets with a pharmacological active status. 4) targets that could be mapped to at least one of our background networks. 5) drugs for which a drug signature could be retrieved from LINCS[35]. After the proximity score calculation, we identified the drugs with a significant score (step 6). For step 5 and 6, venn diagrams indicate the overlap of selected drugs across the three networks.

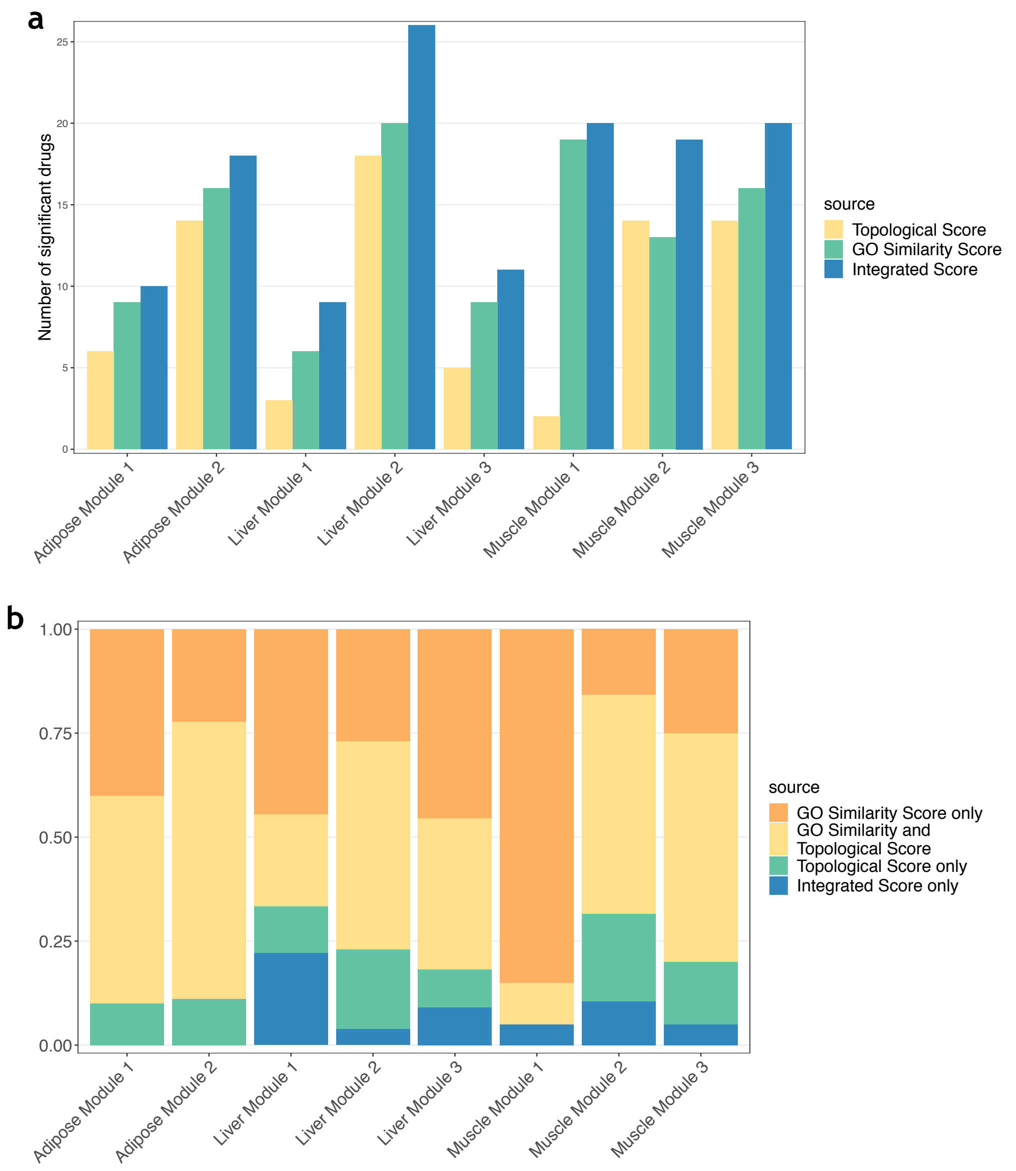

Supplementary Figure 4: Contribution of the topological score and the functional similarity to the final significant results. a Number of significant drugs detected considering only the topological score, only the similarity score, or the integrated score. **b** Relative contribution of the two components of the integrated score to the final predictions. The blue bar indicates drugs only significant when the integrated score was applied.

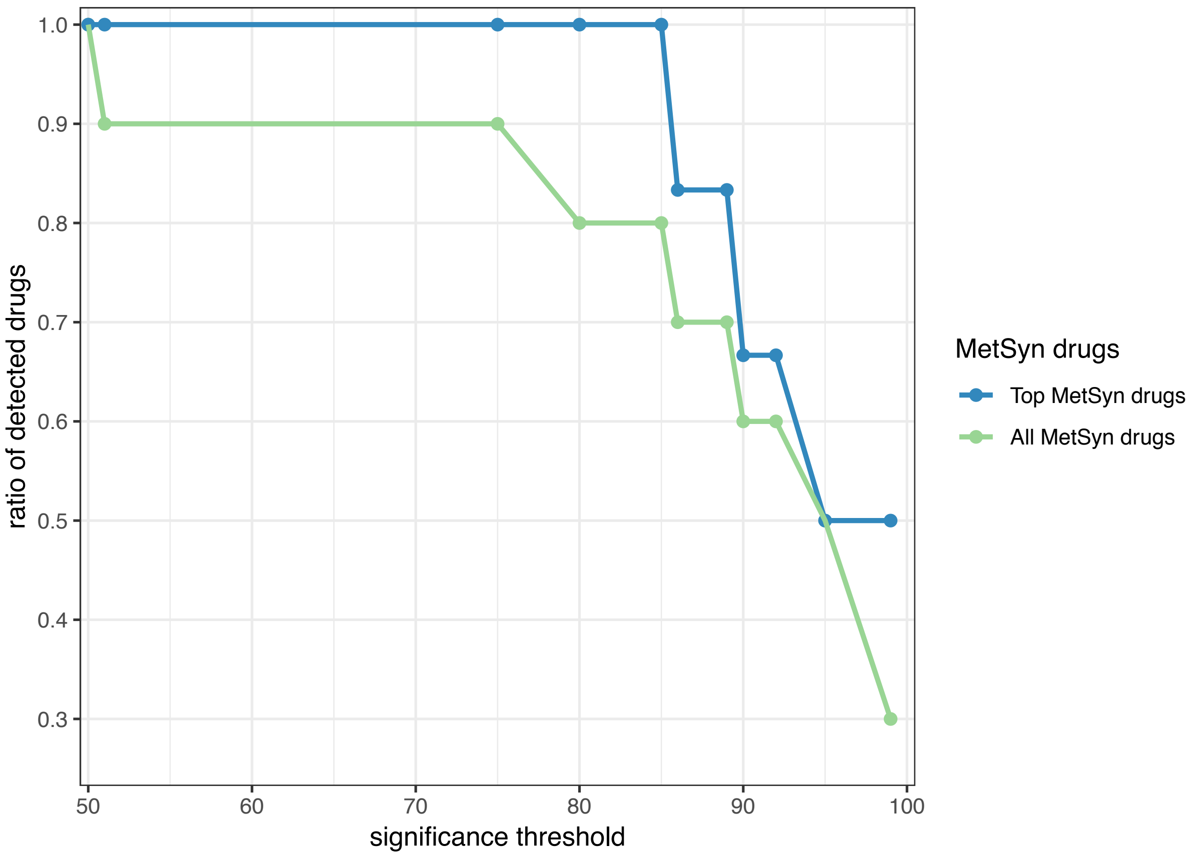

Supplementary Figure 5: Ratio of detected MetSyn drugs at different significance thresholds.

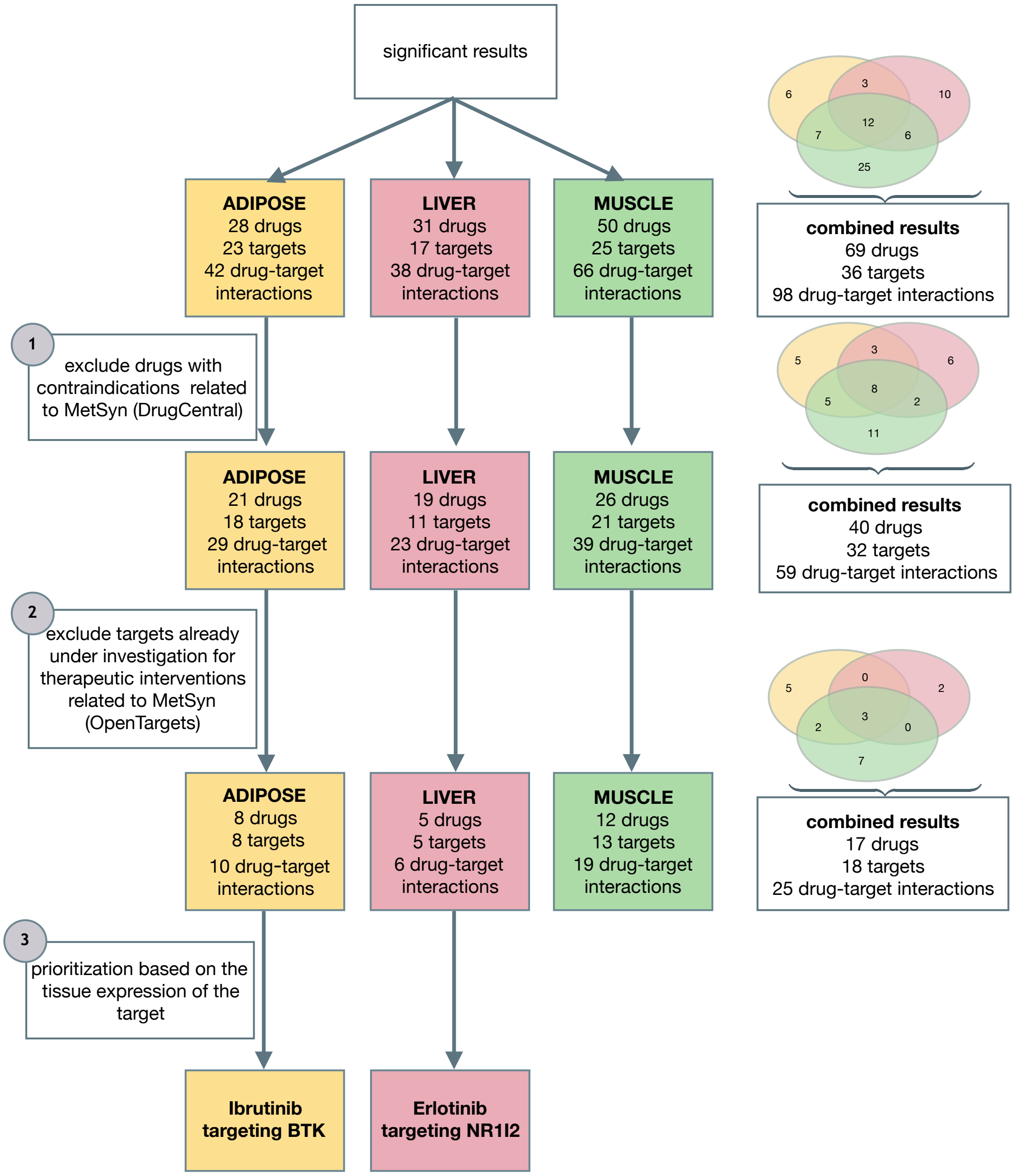

Supplementary Figure 6: Overview of the filtering and prioritization of the significant results. The drugs with a significant score were filtered according to the following steps: 1) drugs with a contraindication related to MetSyn were excluded, 2) drugs with a target that is already under investigation for therapeutic interventions related to MetSyn were excluded, 3) the remaining results were filtered according to the tissue specific expression of the targets. Only the targets of drug-targets interactions with a tissue-specific expression in agreement with MetSyn pathophysiology were kept.

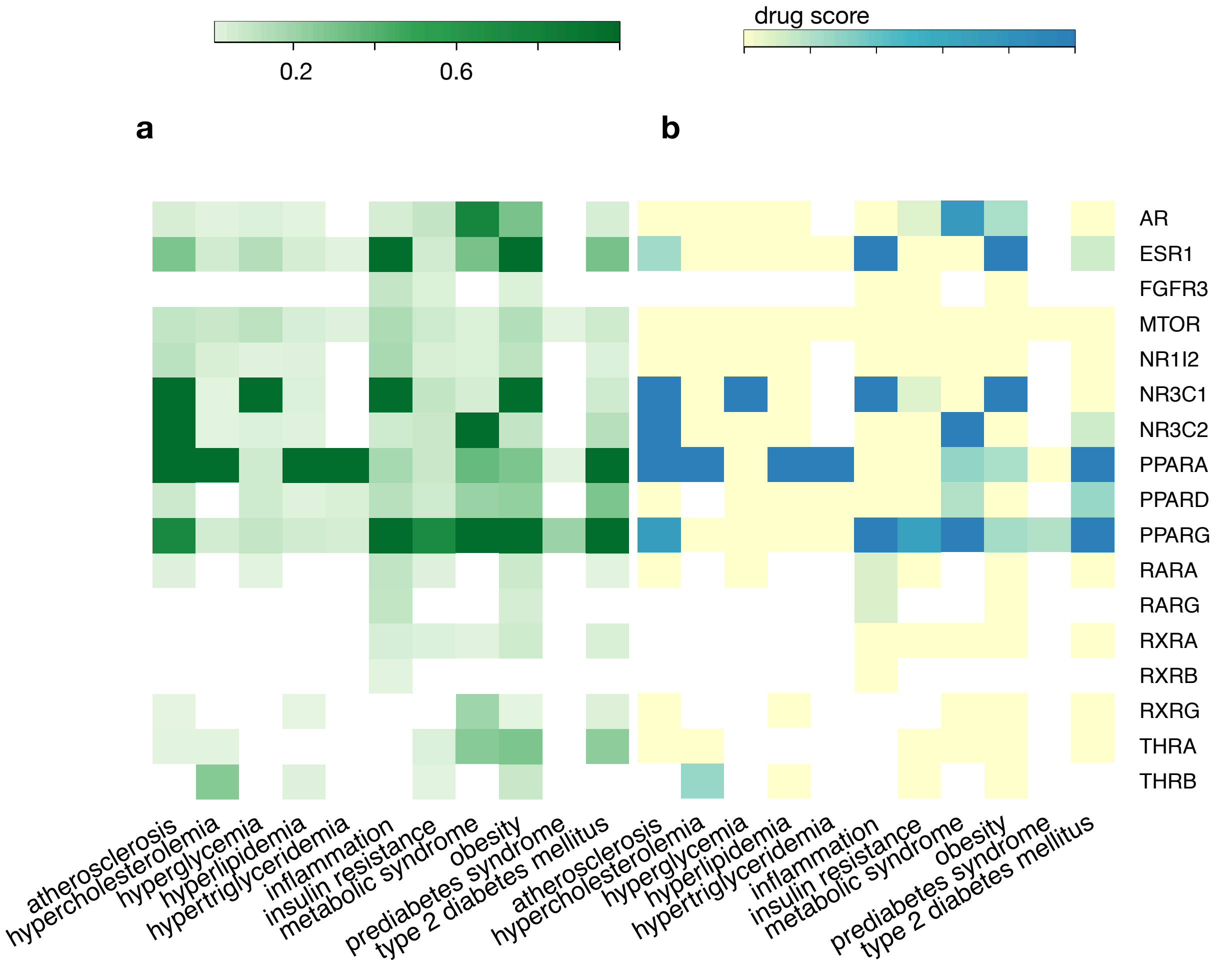

Supplementary Figure 7: Association between the identified active drug targets in the liver network and traits selected to be relevant for MetSyn-related traits based on the association scores provided in by the OpenTargets platform[36].; **a** Heatmap of total association score and **b** heatmap of association score based on ChEMBL[26] information about drugs approved for marketing by FDA or under evaluation in clinical trials. Source data are provided as a Source Data file.

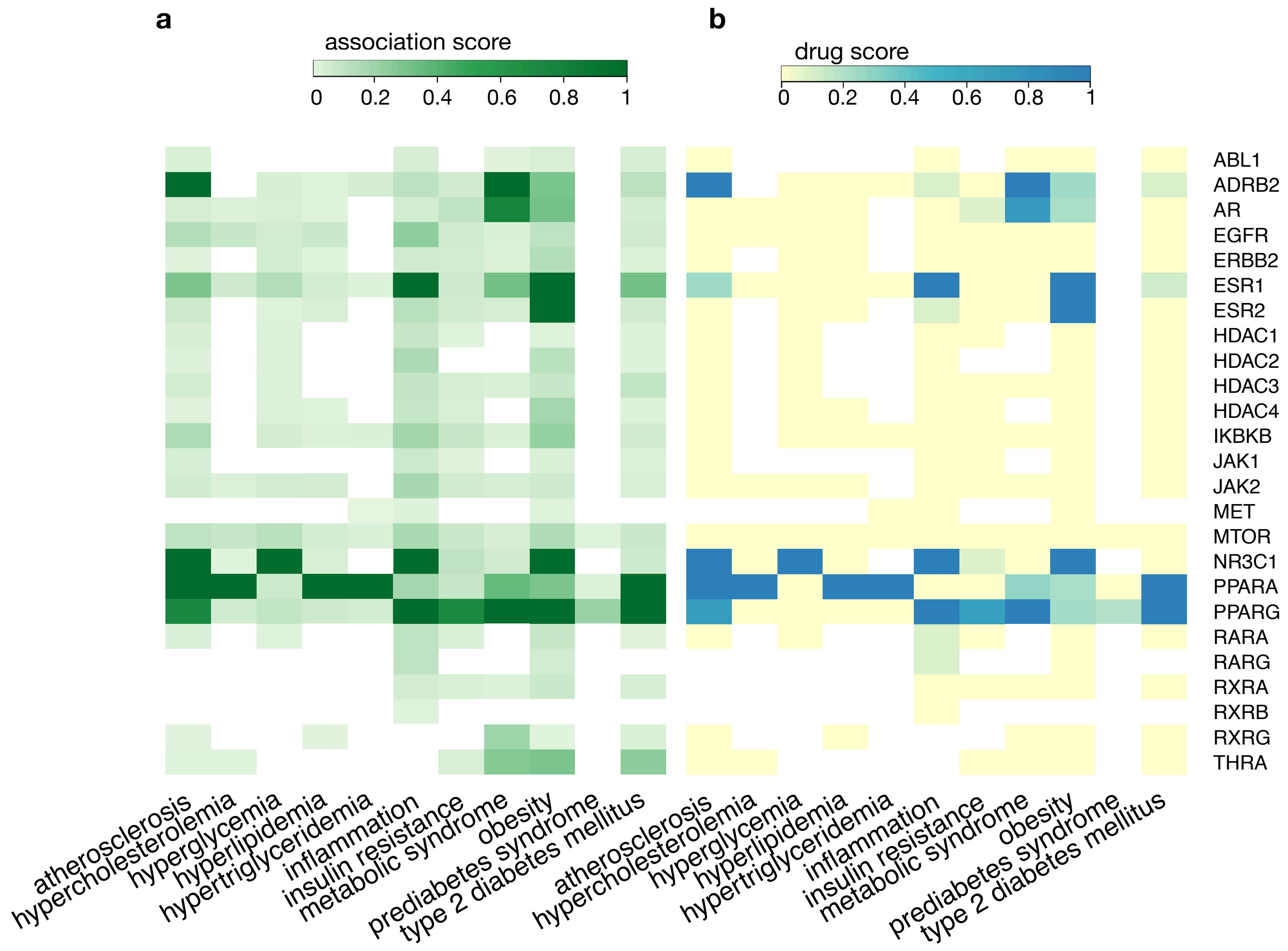

Supplementary Figure 8: Association between the identified active drug targets in the muscle network and traits selected to be relevant for MetSyn-related traits based on the association scores provided in by the OpenTargets platform[36].; a Heatmap of total association score and b heatmap of association score based on ChEMBL[26] information about drugs approved for marketing by FDA or under evaluation in clinical trials. Source data are provided as a Source Data file.

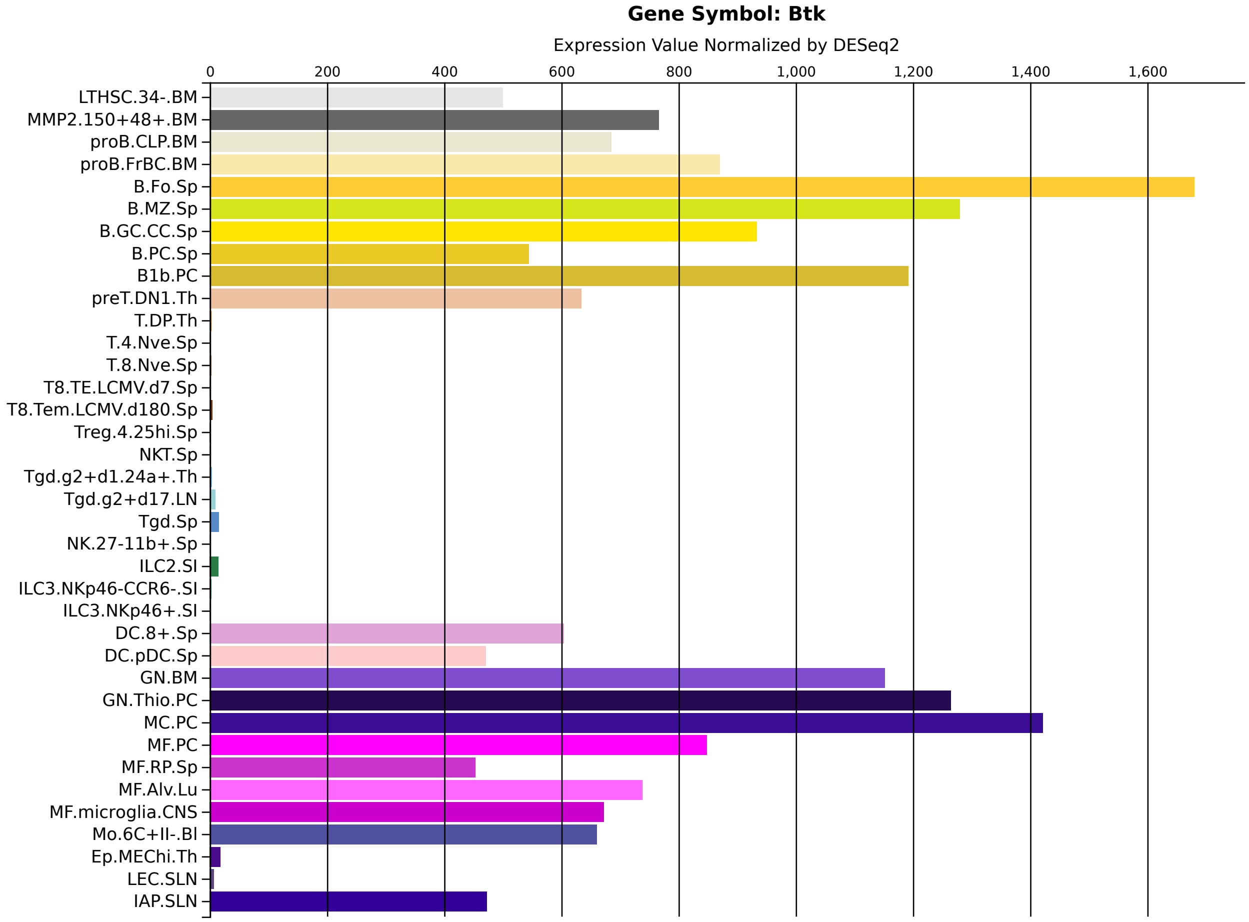

Supplementary Figure 9: Btk expression in immune related cell from ImmGen database.

The bar chart shows Btk levels in derived from RNASeq experiments performed by ImmGen[37].

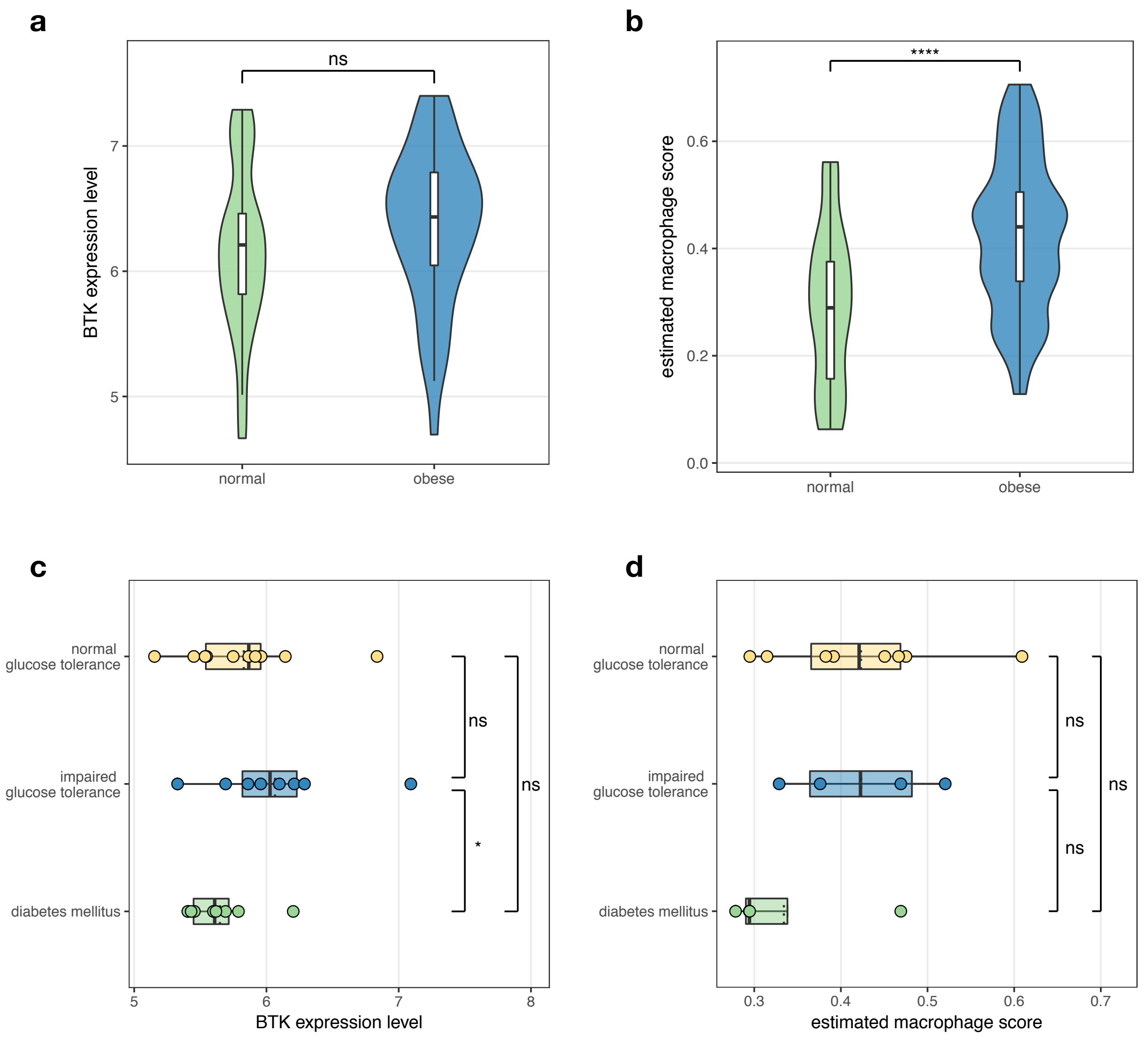

Supplementary Figure 10: BTK expression profiles and macrophages enrichment in human adipose tissue. a BTK expression profiles obtained from the dataset E-MTAB-54[38] in abdominal adipose tissue of obese and normal subjects (Student's t-test). **b** Estimation of macrophages enrichment obtained from the dataset E-MTAB-54[38] in abdominal adipose tissue of obese and normal subjects using CIBERSORT[39]. **c** BTK expression profile obtained from the dataset GSE27951[40] comparing overweight subjects with normal glucose tolerance, impaired glucose tolerance and diabetes mellitus. Student's t-test was used to compare *BTK* expression levels between the different groups. **d** Estimation of macrophages enrichment obtained from the dataset GSE27951[40] comparing overweight subjects with normal glucose tolerance, impaired glucose tolerance and diabetes mellitus using CIBERSORT[39]. Source data are provided as a Source Data file.

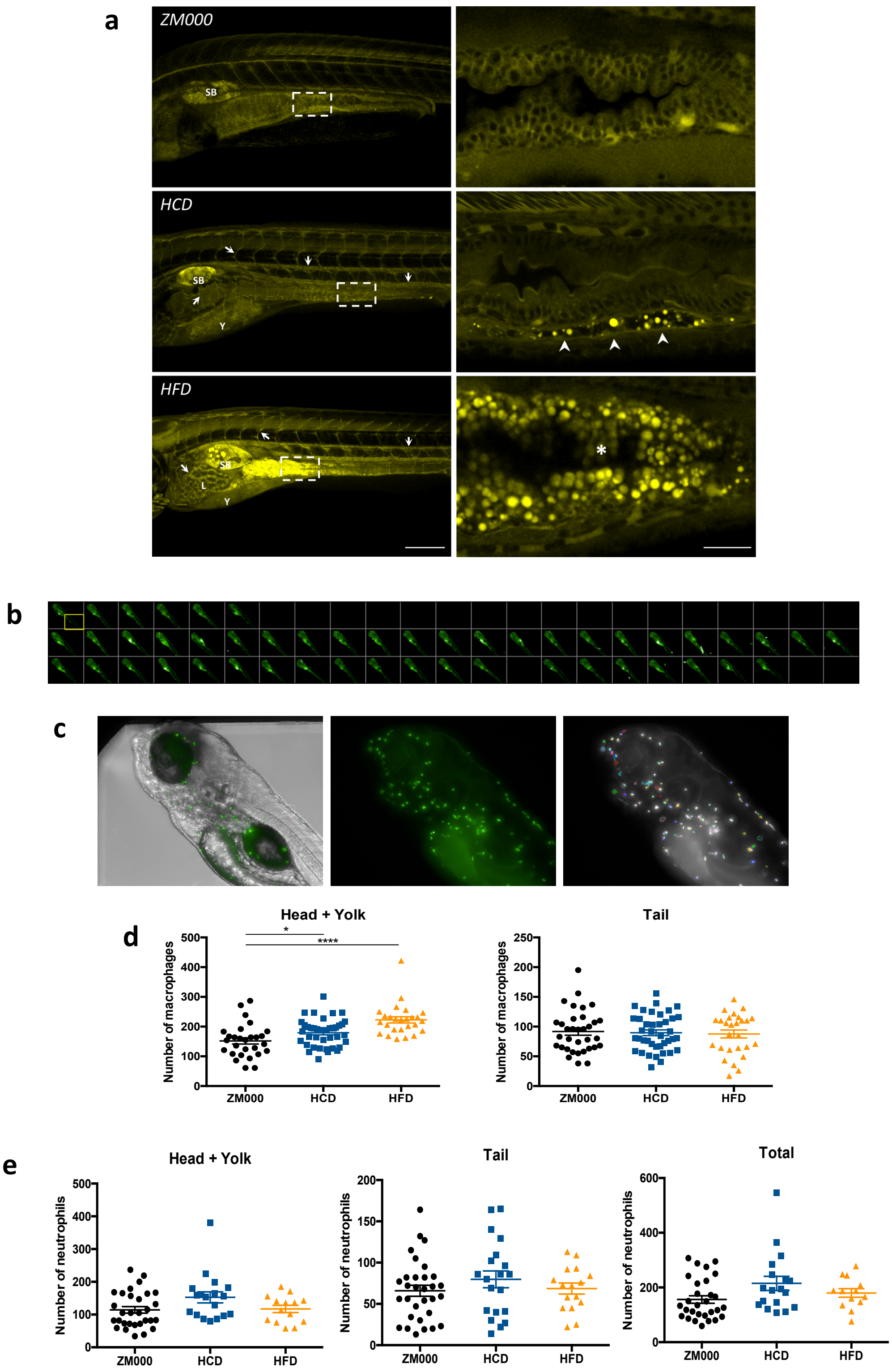
a
ZM000
SB
HCD
SB
Y
HFD
SB
L
Y
*
b
c
d
Head + Yolk
Number of macrophages
500
400
300
200
100
0
*
****
ZM000
HCD
HFD
Tail
Number of macrophages
250
200
150
100
50
0
ZM000
HCD
HFD
e
Head + Yolk
Number of neutrophils
500
400
300
200
100
0
ZM000
HCD
HFD
Tail
Number of neutrophils
200
150
100
50
0
ZM000
HCD
HFD
Total
Number of neutrophils
600
400
200
0
ZM000
HCD
HFD

Supplementary Figure 11: **a** Confocal microscopy live-images of 7 dpf *Casper* larvae stained with Nile Red following indicated diets. i, ii and iii are maximum projections from z-stacks; calibration bar: 200 µm. i', ii', ii", are single plane images zoomed on middle intestine (white boxes in i, ii and iii). Calibration bar: 70 µm for i, ii and iii; 40 µm for i', ii', ii". Arrows point to lipids in blood vessels; arrowheads in ii' point to lipid droplets in the sub-intestinal space; asterisks indicate lipid droplets in intestinal epithelium. SB: swim bladder, L: liver, Y: yolk. **b** Overview of a portion of the 384 plate well used for the screen with oriented and anesthetized larvae. **c** Examples of images acquired and analysed by the operetta system. Embryo region was defined using the eGFP signal. Macrophages or neutrophils (here shown with different pseudo-colors during the process) were selected by automated pattern recognition relying on supervised machine learning (PhenoLOGIC®) and automatically counted. **d** Number of fluorescent macrophages in the head+yolk, and tail region following the indicated diets (n>26). **e** Number of fluorescent neutrophils in the different regions following the indicated treatments (n>14). Data were pooled from 3 or more experiments. HFD: high fat diet; HCD: cholesterol-enriched diet. ***p-value<0.001, *p-value<0.05 (Student's t-test). Source data are provided as Source Data File.

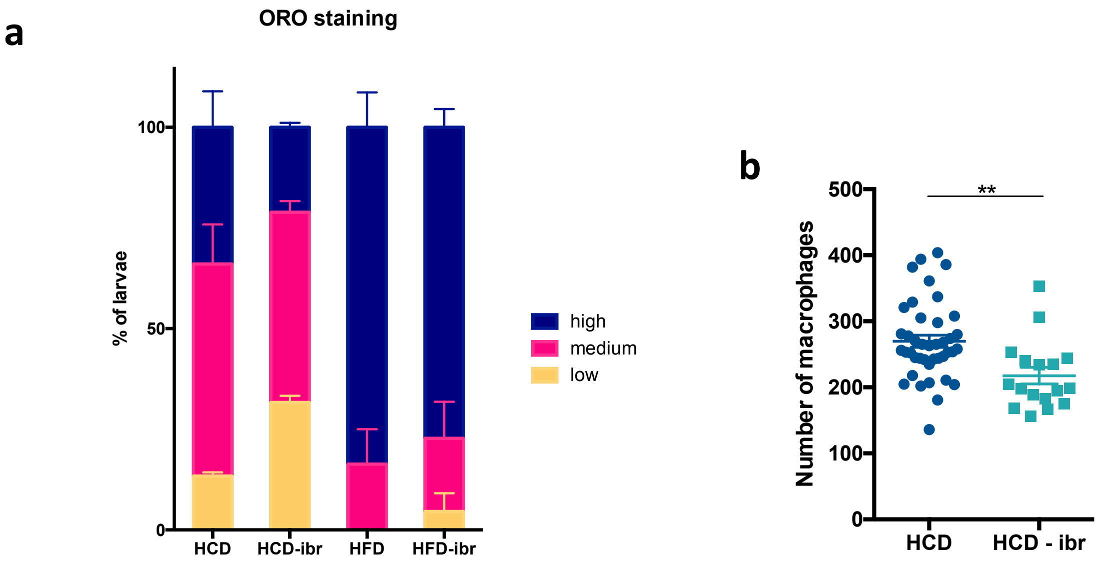

Supplementary figure 12: Effect of ibrutinib treatment on lipid and macrophage accumulation in HFD and HCD fed larve. a Percentage of larvae with high, medium and low lipid accumulation for high-fat (HFD) and high-cholesterol (HCD) diets without or with ibrutinib treatment. There are no significant differences among ibrutinib-treated and untreated groups, despite a slight decrease in lipid accumulation in the treated ones. Data are pooled from 2 experiments (n>12) and the charts show the mean ± the standard error of the mean (SEM). **b** Number of fluorescent macrophages in larvae fed with HCD without and with ibrutinib treatment. Pools from 3 or more experiments, n >16. **p-value<0.01 (Student's t-test). Source data are provided as Source Data file.

Supplementary Tables

Supplementary Table 1: The 15 selected studies used as resource for GWAS summary statistics, obtained from the results of Marbach et al. applying the PASCAL tool[19] (downloaded from http://regulatorycircuits.org/download.html)

TRAIT	FILE	PMID
Coronary artery disease	23_coronary_artery_disease.txt	21378990
Blood pressure (systolic)	22_blood_pressure_systolic.txt	21909115
HDL cholesterol	18_hdl_cholesterol.txt	20686565
LDL cholesterol	19_ldl_cholesterol.txt	20686565
Total cholesterol	20_total_cholesterol.txt	20686565
Triglycerides	21_triglycerides.txt	20686565
Type 2 diabetes	26_type_2_diabetes.txt	22885922
Insulin secretion (CIR)	29_insulin_secretion.txt	24699409
Glucose tolerance	27_2hr_glucose.txt	22885924
Fasting glucose	24_fasting_glucose.txt	22885924
Fasting insulin (BMI-adj.)	32_fasting_insulin.txt	22885924
Glycated hemoglobin	25_glycated_hemoglobin.txt	20858683
Beta-cell function	31_beta-cell_function.txt	20081858
Insulin resistance	30_insulin_resistance.txt	20081858
Fasting proinsulin	28_fasting_proinsulin.txt	21873549

Supplementary Table 2: Contribution of GWAS vs. text mining results to the MetSyn modules.

Network	Module	Module size	Number of disease genes			
			Total	GWAS	Text mining	GWAS and text mining
**Adipose**	1	122	23	0	19	4
	2	51	19	1	10	8
**Liver**	1	17	8	3	1	4
	2	49	21	0	10	11
	3	20	6	3	1	2
**Muscle**	1	299	39	2	31	6
	2	50	17	1	9	7
	3	26	7	0	5	2

Supplementary Table 3: Contribution of regulatory and protein-protein interactions to the final networks.

Network	Number of edges			Number of nodes			
	Total	Regulatory network	PPI	Total	Regulatory network	PPI	Regulatory network and PPI
**Adipose**	9152	255	8900	886	124	661	101
**Liver**	15846	1520	14326	1544	395	983	166
**Muscle**	10555	603	9952	1106	232	741	133

Supplementary Table 4: Contribution of regulatory and protein-protein interactions (network edges) to the edges of the MetSyn modules.

Network	Module	Number of edges		
		Total	Regulatory network	PPI
**Adipose**	1	872	0	872
	2	324	0	324
**Liver**	1	29	29	0
	2	220	8	212
	3	238	0	238
**Muscle**	1	3512	2	3510
	2	248	0	248
	3	202	0	202

Supplementary Table 5: Contribution of regulatory and protein-protein interactions (network nodes) to the nodes of the MetSyn modules.

Network	Module	Number of nodes			
		Total	Regulatory network	PPI	Regulatory network and PPI
**Adipose**	1	122	0	119	3
	2	51	0	44	7
**Liver**	1	17	17	0	0
	2	49	3	37	9
	3	20	0	19	1
**Muscle**	1	299	0	265	34
	2	50	0	38	12
	3	26	0	25	1

Supplementary Table 6: Drugs with indications for MetSyn-related traits affecting adipose tissue.

Drug Name	Indication in DrugBank	Search-term	MoA (ChEMBL)
Atorvastatin	May be used as primary prevention in individuals with multiple risk factors for coronary heart disease (CHD) and as secondary prevention in individuals with CHD to reduce the risk of myocardial infarction (MI), stroke, angina, and revascularization procedures. May be used to reduce the risk of cardiovascular events in patients with acute coronary syndrome (ACS). May be used in the treatment of primary hypercholesterolemia and mixed dyslipidemia, homozygous familial hypercholesterolemia, primary dysbetalipoproteinemia, and/or hypertriglyeridemia as an adjunct to dietary therapy to decrease serum total and low-density lipoprotein cholesterol (LDL-C), apolipoprotein B (apoB), and triglyceride concentrations, while increasing high-density lipoprotein cholesterol (HDL-C) levels.	hypercholes terolemia	HMG-CoA reductase inhibitor
Bezafibrate	For the treatment of primary hyperlipidaemia types IIa, IIb, III, IV and V (Fredrickson classification) corresponding to groups I, II and III of the European Atherosclerosis Society guidelines - when diet alone or improvements in lifestyle such as increased exercise or weight reduction do not lead to an adequate response. Also for the treatment of secondary hyperlipidaemias, e.g. severe hypertriglyceridemias, when sufficient improvement does not occur after correction of the underlying disorder (e.g. diabetes mellitus).	diabetes	Peroxisome proliferator-activated receptor agonist
Clofibrate	For Primary Dysbetalipoproteinemia (Type III hyperlipidemia) that does not respond adequately to diet. This helps control high cholesterol and high triglyceride levels.	hyperlipide mia, High cholesterol	Peroxisome proliferator-activated receptor alpha agonist
Fenofibrate	For use as adjunctive therapy to diet to reduce elevated LDL-C, Total-C,Triglycerides and Apo B, and to increase HDL-C in adult patients with primary hypercholesterolemia or mixed dyslipidemia (Fredrickson Types IIa and IIb)	hypercholes terolemia	Peroxisome proliferator-activated receptor alpha agonist

Gemfibrozil	For treatment of adult patients with very high elevations of serum triglyceride levels (types IV and V hyperlipidemia) who are at risk of developing pancreatitis (inflammation of the pancreas) and who do not respond adequately to a strict diet.	hyperlipidemia	Peroxisome proliferator-activated receptor alpha agonist
Glipizide	For use as an adjunct to diet for the control of hyperglycemia and its associated symptomatology in patients with non-insulin-dependent diabetes mellitus (NIDDM; type II), formerly known as maturity-onset diabetes, after an adequate trial of dietary therapy has proved unsatisfactory.	diabetes, hyperglycemia, NIDDM	Sulfonylurea receptor 1, Kir6.2 blocker
Lovastatin	For management as an adjunct to diet to reduce elevated total-C, LDL-C, apo B, and TG levels in patients with primary hypercholesterolemia and mixed dyslipidemia. For primary prevention of coronary heart disease and to slow progression of coronary atherosclerosis in patients with coronary heart disease.	hypercholesterolemia	HMG-CoA reductase inhibitor
Mifepristone	For the medical termination of intrauterine pregnancy through 49 days' pregnancy. Also indicated to control hyperglycemia secondary to hypercortisolism in adult patients with endogenous Cushing's syndrome who have type 2 diabetes mellitus or glucose intolerance and are not candidates for surgery or have had unsuccessful surgery.	diabetes, hyperglycemia	Glucocorticoid receptor antagonist - Progesterone receptor antagonist
Pioglitazone	Treatment of Type II diabetes mellitus	diabetes	Peroxisome proliferator-activated receptor gamma agonist
Repaglinide	As an adjunct to diet and exercise to improve glycemic control in adults with type 2 diabetes mellitus.	diabetes	Sulfonylurea receptor 1, Kir6.2 blocker

Supplementary Table 7: Tissue expression for the filtered significant targets in the adipose network.

Drug Target	Human protein atlas[41]	GTEx[42]	FANTOM5[43]
BTK	tissue enhanced (tonsil, lymph node)	tissue enhanced (spleen)	tissue enhanced (appendix, spleen, tonsil)
JAK1	expressed in all	expressed in all	expressed in all
PARP1	expressed in all	expressed in all	expressed in all
RARA	expressed in all	expressed in all	expressed in all
RARG	tissue enhanced (skin)	expressed in all	expressed in all
RXRA	mixed	expressed in all	expressed in all
RXRB	mixed	expressed in all	expressed in all
XIAP	expressed in all	expressed in all	expressed in all

Supplementary Table 8: Tissue expression for the filtered significant targets in the liver network.

Drug Target	Human protein atlas[41]	GTEx[42]	FANTOM5[43]
FGFR3	tissue enriched (skin)	tissue enhanced (skin)	tissue enhanced (caudate, hippocampus)
NR1I2	Group enriched (colon, duodenum, gallbladder, liver, rectum, small intestine)	Group enriched (colon, liver, small intestine)	Tissue enhanced (colon, small intestine)
RARA	expressed in all	expressed in all	expressed in all
RXRA	mixed	expressed in all	expressed in all
RXRB	mixed	expressed in all	expressed in all

Supplementary Table 9: Tissue expression for the filtered significant targets in the muscle network.

Drug Target	Human protein atlas	GTEx	FANTOM5
ABL1	expressed in all	expressed in all	expressed in all
ERBB2	expressed in all	expressed in all	expressed in all
HDAC1	expressed in all	expressed in all	expressed in all
HDAC2	expressed in all	expressed in all	expressed in all
HDAC3	expressed in all	expressed in all	expressed in all
HDAC4	mixed	expressed in all	expressed in all
JAK1	expressed in all	expressed in all	expressed in all
JAK2	mixed	expressed in all	expressed in all
MET	expressed in all	mixed	expressed in all
RARA	expressed in all	expressed in all	expressed in all
RARG	tissue enhanced (skin)	expressed in all	expressed in all
RXRA	mixed	expressed in all	expressed in all
RXRB	mixed	expressed in all	expressed in all

Supplementary References

1. Wu, Z., Li, W., Liu, G. & Tang, Y. Network-Based Methods for Prediction of Drug-Target Interactions. *Front. Pharmacol.* **9,** 1–14 (2018).

2. Lotfi Shahreza, M., Ghadiri, N., Mousavi, S. R., Varshosaz, J. & Green, J. R. A review of network-based approaches to drug repositioning. *Brief. Bioinform.* **19,** 878–892 (2017).

3. Pushpakom, S. *et al.* Drug repurposing: progress, challenges and recommendations. *Nat. Rev. Drug Discov.* **18,** 41–58 (2018).

4. Cheng, F. *et al.* Network-based approach to prediction and population-based validation of in silico drug repurposing. *Nat. Commun.* **9,** 2691 (2018).

5. Lamb, J. *et al.* The Connectivity Map: Using Gene-expression signatures to connect small molecules, genes, and disease. *Science.* **313,** 1929–1935 (2006).

6. Lamb, J. The Connectivity map: a new tool for biomedical research. *Nat. Rev. Cancer* **7,** 54–60 (2007).

7. Cheng, F., Zhao, J., Fooksa, M. & Zhao, Z. A network-based drug repositioning infrastructure for precision cancer medicine through targeting significantly mutated genes in the human cancer genomes. *J. Am. Med. Informatics Assoc.* **23,** 681–691 (2016).

8. Lee, S. *et al.* Integrated Network Analysis Reveals an Association between Plasma Mannose Levels and Insulin Resistance. *Cell Metab.* **24,** 172–184 (2016).

9. Lee, S. *et al.* Network analyses identify liver-specific targets for treating liver diseases. *Mol. Syst. Biol.* **13,** 938 (2017).

10. Greene, C. S. *et al.* Understanding multicellular function and disease with human tissue-specific networks. *Nat. Genet.* **47,** 569–576 (2015).

11. Sonawane, A. R. *et al.* Understanding Tissue-Specific Gene Regulation. *Cell Rep.* **21**, 1077–1088 (2017).

12. Cai, C. *et al.* Is human blood a good surrogate for brain tissue in transcriptional studies? *BMC Genomics* **11**, 589 (2010).

13. Winter, E. E., Goodstadt, L. & Ponting, C. P. Elevated rates of protein secretion, evolution, and disease among tissue-specific genes. *Genome Res.* **14,** 54–61 (2004).

14. Lage, K. *et al.* A large-scale analysis of tissue-specific pathology and gene expression of human disease genes and complexes. *PNAS* **105,** 1–6 (2008).

15. Kitsak, M. *et al.* Tissue Specificity of Human Disease Module. *Sci. Rep.* **6,** 1–12 (2016).

16. Sanseau, P. *et al.* Use of genome-wide association studies for drug repositioning. *Nat. Biotechnol.* **30,** 317–320 (2012).

17. Segré, A. V. *et al.* Common inherited variation in mitochondrial genes is not enriched for associations with type 2 diabetes or related glycemic traits. *PLOS Genet.* **6,** e1001058 (2010).

18. Raychaudhuri, S. *et al.* Identifying relationships among genomic disease regions: Predicting genes at pathogenic SNP associations and rare deletions. *PLOS Genet.* **5,** e1000534 (2009).

19. Lamparter, D., Marbach, D., Rueedi, R., Kutalik, Z. & Bergmann, S. Fast and Rigorous Computation of Gene and Pathway Scores from SNP-Based Summary Statistics. *PLOS Comput. Biol.* **12,** e1004714 (2016).

20. Pers, T. H. *et al.* Biological interpretation of genome-wide association studies using predicted gene functions. *Nat. Commun.* **6,** 5890 (2015).

21. de Leeuw, C. A., Mooij, J. M., Heskes, T. & Posthuma, D. MAGMA: Generalized Gene-Set Analysis of GWAS Data. *PLOS Comput. Biol.* **11,** 1–19 (2015).

22. Guney, E., Menche, J., Vidal, M. & Barabási, A.-L. Network-based in silico drug efficacy screening. *Nat. Commun.* **7,** 10331 (2016).

23. Tan, F. *et al.* Drug repositioning by applying 'expression profiles' generated by integrating chemical structure similarity and gene semantic similarity. *Mol. Biosyst.* **10,** 1126–1138 (2014).

24. Vella, D. *et al.* MTGO: PPI Network Analysis Via Topological and Functional Module Identification. *Sci. Rep.* **8,** 1–13 (2018).

25. Wishart, D. S. *et al.* DrugBank 5.0: A major update to the DrugBank database for 2018. *Nucleic Acids Res.* **46,** D1074–D1082 (2018).

26. Davies, M. *et al.* ChEMBL web services: streamlining access to drug discovery data and utilities. *Nucleic Acids Res.* **43,** W612–W620 (2015).

27. Yamada, Y. *et al.* Atorvastatin reduces cardiac and adipose tissue inflammation in rats with metabolic syndrome. *Int. J. Cardiol.* **240,** 332–338 (2017).

28. Pengde, K., Fuxing, P., Bin, S., Jing, Y. & Jingqiu, C. Lovastatin inhibits adipogenesis and prevents osteonecrosis in steroid-treated rabbits. *Jt. Bone Spine* **75,** 696–701 (2008).

29. Nakata, M. *et al.* Effects of statins on the adipocyte maturation and expression of glucose transporter 4 (SLC2A4): Implications in glycaemic control. *Diabetologia* **49,** 1881–1892 (2006).

30. Sola, D. *et al.* Sulfonylureas and their use in clinical practice. *Arch. Med. Sci.* **11,** 840–848 (2015).

31. Thulé, P. M. & Umpierrez, G. Sulfonylureas: a new look at old therapy. *Curr. Diab. Rep.* **14,** 473 (2014).

32. MacArthur, J. *et al.* The new NHGRI-EBI Catalog of published genome-wide association studies (GWAS Catalog). *Nucleic Acids Res.* **45,** D896–D901 (2017).

33. Hsu, F. *et al.* The UCSC known genes. *Bioinformatics* **22,** 1036–1046 (2006).

34. Roadmap Epigenomics Consortium *et al.* Integrative analysis of 111 reference human epigenomes. *Nature* **518,** 317–329 (2015).

35. Subramanian, A. *et al.* A Next Generation Connectivity Map: L1000 Platform and the First 1,000,000 Profiles. *Cell* **171,** 1437–1452 (2017).

36. Koscielny, G. *et al.* Open Targets: A platform for therapeutic target identification and validation. *Nucleic Acids Res.* **45,** D985–D994 (2017).

37. Heng, T. S. P. & Painter, M. W. The Immunological Genome Project: networks of gene expression in immune cells. *Nat. Immunol.* **9,** 1091–1094 (2008).

38. Drong, A. W. *et al.* The Presence of Methylation Quantitative Trait Loci Indicates a Direct Genetic Influence on the Level of DNA Methylation in Adipose Tissue. *PLoS One* **8,** (2013).

39. Newman, A. M. *et al.* Robust enumeration of cell subsets from tissue expression profiles. *Nat. Methods* **12,** 453–457 (2015).

40. Keller, P. *et al.* Gene-chip studies of adipogenesis-regulated microRNAs in mouse primary adipocytes and human obesity. *BMC Endocr. Disord.* **11,** 7 (2011).

41. Uhlen, M. *et al.* Tissue-based map of the human proteome. *Science.* **347,** 1–9 (2015).

42. The GTEx Consortium. The Genotype-Tissue Expression (GTEx) pilot analysis: Multitissue gene regulation in humans. *Science.* **348,** 648–660 (2015).

43. Lizio, M. *et al.* Gateways to the FANTOM5 promoter level mammalian expression atlas. *Genome Biol.* **16,** 1–14 (2015).
