# Supplementary material for: A network-based approach to identify deregulated pathways and drug effects in metabolic syndrome
Source: Nat Commun. 2019 Nov 18;10:5215. doi: 10.1038/s41467-019-13208-z (PMC6861239; doi:10.1038/s41467-019-13208-z)
Supplement: Supplementary file 3 — Description of Additional Supplementary Files [file 41467_2019_13208_MOESM3_ESM.pdf]

Description of Additional Supplementary Files

File Name: Supplementary Data 1
Description: SNPs extracted from the GWAS catalog that have been identified as associated with Metabolic Syndrome.

File Name: Supplementary Data 2
Description: Pathways enriched in MetSyn genes identified using EnrichR.

File Name: Supplementary Data 3
Description: Genes associated with MetSyn that are located in significant pathways. For each gene the source (text-mining, GWAS, or both) is reported.

File Name: Supplementary Data 4
Description: Interactions included in the 3 networks (adipose, liver, muscle). For each couple of interacting genes/proteins the information source (HIPPIE protein-protein interaction network or Regulatory network) is reported.

File Name: Supplementary Data 5
Description: Significant pathways for all the significant network modules related to the 3 tissue-specific networks.

File Name: Supplementary Data 6
Description: List of drugs with a significant score. For each drug is annotated the target, the action and the indication reported in DrugBank database, the score, the network module where the target was identified, the presence of MetSyn-related side effect (from DrugCentral db), and the existence of studies that already investigated the drug for MetSyn (according to OpenTargets db).

File Name: Supplementary Data 7
Description: Side effects associated to the drugs with a significant score (from Drug Central db).
