# Supplementary material for: A network-based approach to identify deregulated pathways and drug effects in metabolic syndrome
Source: Nat Commun. 2019 Nov 18;10:5215. doi: 10.1038/s41467-019-13208-z (PMC6861239; doi:10.1038/s41467-019-13208-z)
Supplement: Supplementary file 11 — Reporting Summary [file 41467_2019_13208_MOESM11_ESM.pdf]

nature research

Corresponding author(s): Silvia Parolo, Enrico Domenici, Corrado Priami

Last updated by author(s): Oct 17, 2019

# Reporting Summary

Nature Research wishes to improve the reproducibility of the work that we publish. This form provides structure for consistency and transparency in reporting. For further information on Nature Research policies, see Authors & Referees and the Editorial Policy Checklist.

## Statistics

For all statistical analyses, confirm that the following items are present in the figure legend, table legend, main text, or Methods section.

| n/a | Confirmed | |
|---|---|---|
| ☐ | ☒ | The exact sample size (*n*) for each experimental group/condition, given as a discrete number and unit of measurement |
| ☐ | ☒ | A statement on whether measurements were taken from distinct samples or whether the same sample was measured repeatedly |
| ☐ | ☒ | The statistical test(s) used AND whether they are one- or two-sided<br>*Only common tests should be described solely by name; describe more complex techniques in the Methods section.* |
| ☒ | ☐ | A description of all covariates tested |
| ☐ | ☒ | A description of any assumptions or corrections, such as tests of normality and adjustment for multiple comparisons |
| ☐ | ☒ | A full description of the statistical parameters including central tendency (e.g. means) or other basic estimates (e.g. regression coefficient) AND variation (e.g. standard deviation) or associated estimates of uncertainty (e.g. confidence intervals) |
| ☐ | ☒ | For null hypothesis testing, the test statistic (e.g. *F*, *t*, *r*) with confidence intervals, effect sizes, degrees of freedom and *P* value noted<br>*Give P values as exact values whenever suitable.* |
| ☒ | ☐ | For Bayesian analysis, information on the choice of priors and Markov chain Monte Carlo settings |
| ☒ | ☐ | For hierarchical and complex designs, identification of the appropriate level for tests and full reporting of outcomes |
| ☒ | ☐ | Estimates of effect sizes (e.g. Cohen's *d*, Pearson's *r*), indicating how they were calculated |

*Our web collection on statistics for biologists contains articles on many of the points above.*

## Software and code

Policy information about availability of computer code

| | |
|---|---|
| Data collection | No software was used to collect the data used in this study with the exception of LINCS data that were retrieved from LINCS API with a custom python script |
| Data analysis | Data analysis was performed using R programming (version 3.4.3). Platform: x86_64-pc-linux-gnu (64-bit), running under: Debian GNU/ Linux 9 |

For manuscripts utilizing custom algorithms or software that are central to the research but not yet described in published literature, software must be made available to editors/reviewers. We strongly encourage code deposition in a community repository (e.g. GitHub). See the Nature Research guidelines for submitting code & software for further information.

## Data

Policy information about availability of data

All manuscripts must include a data availability statement. This statement should provide the following information, where applicable:

- Accession codes, unique identifiers, or web links for publicly available datasets
- A list of figures that have associated raw data
- A description of any restrictions on data availability

The list of SNPs associated with metabolic syndrome analyzed in this study was obtained from the NHGRI-EBI GWAS catalog (https://www.ebi.ac.uk/gwas/). The gene regulatory networks were retrieved from regulatory circuits (http://regulatorycircuits.org/download.html) portal. The protein-protein interaction network was downloaded from HIPPIE (http://cbdm-01.zdv.uni-mainz.de/~mschaefer/hippie/index.php ). The drug expression profiles were retrieved from the Library of Integrated Cellular Signatures (https://clue.io/) portal. The gene expression datasets were downloaded from NCBI GEO (https://www.ncbi.nlm.nih.gov/geo/) database and from EMBL-EBI ArrayExpress (https://www.ebi.ac.uk/arrayexpress/). The list of drugs and targets was downloaded from DrugBank (https://www.drugbank.ca/). The data about the drug targets were downloaded from Open Targets (https://www.opentargets.org/) platform. The data about the side effects were downloaded from Drug central (http://drugcentral.org) platform.
The authors declare that the data produced by the analyses in this manuscript are available within the article and its Supplementary Information files. The source

data underlying Figs 6a-b, 7a-d, 8c, 8e-h, 9b, 9d-e and Supplementary Figs 7a-b, 8a-b, 10a-d, 11d-e, 12a-b are provided as a Source Data file.

# Field-specific reporting

Please select the one below that is the best fit for your research. If you are not sure, read the appropriate sections before making your selection.

☒ Life sciences ☐ Behavioural & social sciences ☐ Ecological, evolutionary & environmental sciences

For a reference copy of the document with all sections, see nature.com/documents/nr-reporting-summary-flat.pdf

# Life sciences study design

All studies must disclose on these points even when the disclosure is negative.

| | |
|---|---|
| Sample size | Sample size was >12 in all cases. This size is considered sufficient based on the low variability |
| Data exclusions | No data were excluded |
| Replication | All replication were successful |
| Randomization | Randomization was performed. |
| Blinding | At least one Investigator was blinded to group allocation |

# Reporting for specific materials, systems and methods

We require information from authors about some types of materials, experimental systems and methods used in many studies. Here, indicate whether each material, system or method listed is relevant to your study. If you are not sure if a list item applies to your research, read the appropriate section before selecting a response.

## Materials & experimental systems

| n/a | Involved in the study | |
|---|---|---|
| ☒ | ☐ | Antibodies |
| ☒ | ☐ | Eukaryotic cell lines |
| ☒ | ☐ | Palaeontology |
| ☐ | ☒ | Animals and other organisms |
| ☒ | ☐ | Human research participants |
| ☒ | ☐ | Clinical data |

## Methods

| n/a | Involved in the study | |
|---|---|---|
| ☒ | ☐ | ChIP-seq |
| ☒ | ☐ | Flow cytometry |
| ☒ | ☐ | MRI-based neuroimaging |

## Animals and other organisms

Policy information about studies involving animals; ARRIVE guidelines recommended for reporting animal research

| | |
|---|---|
| Laboratory animals | Danio Rerio |
| Wild animals | No |
| Field-collected samples | No |
| Ethics oversight | All animal experiments were performed in accordance with European guidelines and regulations, and were approved by the ethic board of the University of Trento. Approval for breeding was granted by the local government (Città di Trento, C_L378/ S022/103359/03.06.2015 to Università di Trento). |

Note that full information on the approval of the study protocol must also be provided in the manuscript.
